# Trend in active transportation to school among Swiss school children and its associated factors: three cross-sectional surveys 1994, 2000 and 2005

**DOI:** 10.1186/1479-5868-7-28

**Published:** 2010-04-15

**Authors:** Leticia Grize, Bettina Bringolf-Isler, Eva Martin, Charlotte Braun-Fahrländer

**Affiliations:** 1 Swiss Tropical and Public Health Institute, Socinstrasse 57, 4002 Basel, Switzerland; 2 University of Basel, Basel, Switzerland; 3 Federal Institute of Sports, 2532 Magglingen, Switzerland; 4 Institute of Social and Preventive Medicine, Zurich, Switzerland

## Abstract

**Background:**

Giving the rising trend in childhood obesity in many countries including Switzerland, strategies to increase physical activity such as promoting active school travel are important. Yet, little is known about time trends of active commuting in Swiss schoolchildren and factors associated with changes in walking and biking to school.

**Methods:**

Between 1994 and 2005, information about mobility behaviour of children aged 6-14 years was collected within three Swiss population based national travel behaviour surveys. Mode of transport to school was reported for 4244 children. Weighted multivariate logistic regression analyses were used to assess active school travel time trends and their influencing factors.

**Results:**

More than 70% of Swiss children walked or biked to school. Nevertheless, the proportion of children biking to school decreased (p = 0.05, linear trend), predominately in urban areas, and motorized transportation increased since 1994 (p = 0.02). Distance to school did not change significantly over time but availability of bikes decreased (p < 0.001) and number of cars per household increased (p < 0.001). The association between survey year and bike use was significantly modified by living in an urban area (OR (95%CI): 1.0, 0.63 (0.44-0.90), 0.71 (0.49-1.03), respectively for 1994, 2000 and 2005) and by distance to school (OR (95%CI): 1.0, 0.65 (0.40-1.05), 0.50 (0.23-0.79) for the same years and for children who lived more than a mile away from school).

**Conclusions:**

Programs to encourage safe biking and to limit car use as mode of transport to school are warranted to stop this trend.

## Background

Walking, bicycling, or using other modes of active travel to school provides an opportunity to incorporate regular physical activity into the lives of today's children [1]. Studies have shown active transportation to be associated with increased daily energy expenditure and increased cardiovascular fitness when compared to travelling by car [2,3]. Given the rising trend of childhood obesity in many countries including Switzerland [4], strategies to increase children's physical activity are of great public health relevance.

The proportion of children walking and cycling to school varies considerably across countries [5]. A recent analysis of personal and environmental factors associated with active commuting to school in Switzerland has shown that the proportion of active commuters is still high (77.8%) but varies within the country [6]: for single trips to school, French speaking children were significantly more likely to be driven by car than their German speaking peers. In line with other studies [7,8], the Swiss study found major road crossings and distance to school to act as the main barriers to walking or biking to school.

Despite the potential health benefits of active commuting, studies from Australia, Canada, the US and the United Kingdom reported declining proportions of children walking and biking to school over the past decades [9-15]. However, little is known about the details of this decline as most of the known barriers to walking have not been studied over time. A recent analysis of the US National Personal Transportation Survey data has suggested that distance to school has increased over time and may account for half of the decline in active transportation to school [13].

In Switzerland, the problem is widely discussed in mass media and among public health professionals. Initiatives such as KidsWalk-to-School programs (Pédibus [16]) have been implemented in many regions of Switzerland but so far no analyses of time trends have been published in the peer-reviewed literature. Previous descriptive analyses of the 6 to 20 years old participating in the presented surveys showed that although the degree of motorization among adolescents declined, there was a marked drop in bicycle use. An increase in the proportion of children driven to school was also observed [17].

The present study fills this gap by analyzing the data from three consecutive Swiss Transport Surveys conducted in 1994, 2000 and 2005. The primary aim of the study was to document time trends of active transportation to school in children and young adolescents (6 to 14 years old) and to evaluate if sociodemographic and environmental factors are associated with a change in walking and biking to school.

## Methods

The Swiss Microcensus on Travel Behaviour is a population-based national survey conducted every five years since 1974 by the Swiss Federal Statistical Office and the Federal Office for Spatial Development. Since 1994, mobility behaviour of children aged 6 years and older is included in the survey. The present analyses are thus based on the travel surveys from 1994, 2000 and 2005.

The surveys collected information by one-day retrospective telephone interviews on trips undertaken by members of a selected household on a randomly assigned survey day. Interviews were distributed over the whole year. Household members were asked to provide information on mode of transportation for all stages of a trip (e.g. walking from home to the bus stop, taking the bus and walking from bus stop to school). In addition, the purpose of a trip and the travel distance were enquired, and data on the demographic characteristics of the household members were collected. Proxy respondents were interviewed if children were below the age of 14 years in 2005 and 60% of the 10-14 years old responded themselves in the earlier surveys.

The participation rate of the selected persons was 69.9% (18020), 70.5% (29407) and 64.7% (33390) in 1994, 2000 and 2005, respectively. About 10% of the persons refused to participate; most others could not be reached by phone or did not speak one of the local languages.

The present analyses are restricted to schoolchildren aged 6-14 years, to trips to and from school and to days on which children actually went to school, and include a total of 4244 children.

Because different modes of transport could be used for different stages of a trip, we defined the following mutually exclusive categories of mode of transportation: 'foot only' (all stages of a trip to and from school performed by foot), 'bike' (including bike-only trips and combinations of walking and biking), 'public transport' (all combinations of public transportation with walking and/or biking, excluding combinations with car use), 'any car use' (car-only trips as well as all combinations including a stage travelled by car).

In addition, we defined 'active transportation to school' as a combination of trips either by 'foot only' or by 'bike'.

### Statistical analysis

Statistical analysis was performed with SAS version 9.1 (Cary, N.C.: SAS Institute 2002). Sample weighted prevalence of the four different modes of transport to school (by foot only, bike use, public transport use and any car use) were calculated. Differences among sample weighted proportions of characteristics of the study population in the different surveys were tested using the Rao-Scott χ^2 ^test. Weights accounting for age, sex, nationality, region, day of the week and season were provided by the Swiss Federal Office of Statistics [18]. Unadjusted trends were calculated using logistic regression models for discrete response survey data.

To determine the relative change over time with a certain mode of transport to school and the factors which could influence this change, multiple logistic regression models for survey data were used. Models included survey year, socio-demographic characteristics (sex, age, nationality, region type and language) and environmental factors (distance to school, number of cars at home and bike availability). Interactions between the survey years and other factors were tested.

## Results

**Figure 1:**
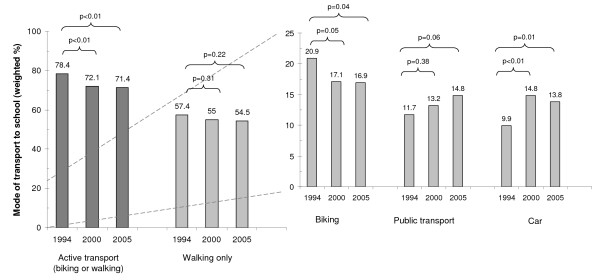
**Weighted prevalence of mode of transport to school according to survey year**.

Figure 1 displays the weighted proportion of children who actively commuted to school (walking and/or biking) by year of survey. This figure also shows the specific proportion of children who walked, biked, used pubic transport or rode a car. The proportion of children actively commuting to school significantly decreased from 78.4% in 1994 to 72.1% in 2000 and 71.4% in 2005 (p = 0.002, linear trend). The decrease was mainly due to a reduction of bike use over time (p = 0.05, linear trend), and less pronounced to a reduction in walking to school. Concomitantly, some increase was observed for using public transportation and the percentage of children riding a car significantly increased (p = 0.02, linear trend). This increase was mainly due to car use for some stages of a trip, as the weighted percentage of children driven in all trips to and from school was only 3.1%, 4.9%, and 2.8% in 1994, 2000 and 2005, respectively. Some changes in the socio-demographic composition of the sample were also noted over time (table 1). Between 1994 and 2005 the proportion of German speaking children and of children living in urban areas significantly decreased. Across all three surveys, the proportion of actively commuting children was significantly different among the three language regions of the country (80.2%, 55.8% and 51.7% (p < 0.001) in the German, the French, and the Italian speaking part, respectively).

**Table 1:** Characteristics of the study population over time

		1994 (n = 956)	2000 (n = 1535)	2005 (n = 1753)	Test for linear trend p-value_wt_
			
Characteristic		%_wt_	%_wt_	%_wt_	
**Sociodemographic**					
Sex:	boys	53.5	52.8	52.8	0.8081
Age:	mean_wt _± se_wt_	10.2 ± 0.10	10.1 ± 0.08	10.2 ± 0.08	0.6582
Nationality:	Swiss	81.2	81.6	78.9	0.2590
Area type:	urban	63.8	59.4	57.0	0.0042
Regional language:	German	75.6	72.3	70.0	0.0058
	French	21.4	23.4	24.8	
	Italian	3.0	4.3	5.2	
**Environmental**					
Distance to school:	in Km mean_wt _± se_wt_	2.2 ± 0.19	2.5 ± 0.25	2.4 ± 0.14	0.5721
	within ≤ 1 Km	60.7	59.7	55.2	0.0155
	within ≤ 1 mile^†^	70.9	69.1	67.6	0.1342
Number of cars at home:	none	9.0	6.2	5.8	< 0.0001
	1	64.5	52.6	52.5	
	2	22.9	35.9	37.1	
	≥ 3	3.0	5.3	4.6	
Bicycle availability:	always	91.1	86.2	85.1	0.0008
	need to ask	3.4	3.8	9.1	
	Never	5.5	10.0	5.8	

^† ^1 mile = 1.609 Km

wt = sample weighted

Table 1 also shows the distribution of environmental and household characteristics potentially influencing the development of active commuting over time. The mean distance to school did not change significantly and across all three surveys a large proportion of children lived within one mile from school (70.9%, 69.1% and 67.6% in 1994, 2000, and 2005, respectively). Yet the proportion of children living within 1 km significantly decreased (60.7%, 59.7% and 55.2%; p = 0.0155). The number of cars per household significantly increased over time (p < 0.0001), whereas bike availability significantly decreased (p < 0.001).

To evaluate whether any of these environmental factors influenced the trend of bike and car use over time, multiple regression models were run first adjusting for socio-demographic variables and then additionally for environmental variables. The results are summarized in table 2.

**Table 2:** Factors associated with bike and car use as mode of transport to school during 1994-2005

		Bike use		Car use	
			
		Model adjusted for socio-demographic factors	Model adjusted for socio-demographic +environmental factors	Model adjusted for socio-demographic factors	Model adjusted for socio-demographic +environmental factors
			
Factor		OR_wt_ (95% CI_wt_)	OR_wt_ (95% CI_wt_)	OR_wt_ (95% CI_wt_)	OR_wt_ (95% CI_wt_)
Survey year:	1994	1.0	1.0	1.0	1.0
	2000	0.79 (0.61-1.03)	0.83 (0.63-1.09)	**1.53** (1.13-2.07)	**1.45** (1.06-1.99)
	2005	**0.75** (0.57-0.98)	0.81 (0.61-1.07)	**1.40** (1.03-1.89)	1.30 (0.94-1.79)
Sex:	girls	1.0	1.0	1.0	1.0
	Boys	1.19 (0.96-1.48)	1.17 (0.94-1.46)	0.95 (0.76-1.18)	0.96 (0.76-1.21)
Age:	6 to 9	1.0	1.0	1.0	1.0
	10 to 12	**4.54** (3.37-6.10)	**4.26** (3.15-5.75)	**0.58** (0.45-0.75)	**0.51** (0.39-0.67)
	13 to 14	**9.67** (7.16-13.10)	**9.84** (7.21-13.42)	**0.56** (0.42-0.75)	**0.33** (0.22-0.47)
Nationality:	non-Swiss	1.0	1.0	1.0	1.0
	Swiss	1.16 (0.82-1.64)	1.06 (0.74-1.51)	1.22 (0.89-1.67)	1.12 (0.81-1.54)
Region:	non-urban	1.0	1.0	1.0	1.0
	urban	**0.69** (0.55-0.86)	**0.70** (0.56-0.87)	1.09 (0.87-1.38)	1.18 (0.92-1.51)
Language:	German	1.0	1.0	1.0	1.0
	French	**0.18** (0.12-0.25)	**0.19** (0.13-0.270)	**2.51** (1.99-3.17)	**2.18** (1.71-2.79)
	Italian	**0.42** (0.21-0.82)	**0.45** (0.23-0.86)	**3.42** (2.24-5.22)	**3.20** (2.05-5.00)
Distance to school (Km):		-	**0.93** (0.89-0.96)	-	**1.16** (1.12-1.21)
Cars at home:	None or 1 car	-	1.0	-	1.0
	2 or more cars	-	1.10 (0.87-1.39)	-	**1.72** (1.35-2.18)
Bike availability:	always	-	1.0	-	1.0
	not always	-	**0.13** (0.07-0.23)	-	1.09 (0.77-1.53)

Across all three surveys, older children were significantly more likely to bike to school, whereas children living in urban areas, and French and Italian speaking children were less likely to bike. Adjusting for these sociodemographic variables did not explain the decrease in bike use over time (unadjusted OR (95% CI): 1.0, 0.78 (0.61 - 1.00), 0.77 (0.60 - 0.90), and adjusted for sociodemographics: 1.0, 0.79 (0.61 - 1.03), 0.75 (0.57 - 0.98), where the 1994 survey was the reference). Adding distance to school or number of cars at home did not change the trend of biking (adjusted for sociodemographics) but addition of bike availability did (1.0, 0.84 (0.65 - 1.10), 0.81 (0.61 - 1.06); respectively for 1994, 2000 and 2005).

Across all surveys, driving children to school by car was significantly less common in older children but much more popular among French and Italian speaking children. Car use as mode of transportation to school increased significantly with distance from school and when families owned two or more cars. The association of car use with survey year remained significant when adjusting for sociodemographic factors and was attenuated by the additional adjustment for environmental variables (table 2). The odds ratio of using a car remained significantly elevated for the 2000 survey as compared to 1994 (OR:1.45, 95%CI:1.06 - 1.99).

We then evaluated whether variables included in the multiple regression models significantly modified the association between survey year and car and bike use as mode of transport to school. No significant interactions were observed for car use and survey year for any of the tested variables. Yet, the association between survey year and bike use was significantly modified by urban living (OR:0.58, 95%CI: 0.34 - 1.00, for the interaction of urban area and survey year 2000) and distance to school (OR:0.93, 95%CI: 0.87 - 1.00 and OR:0.88, 95%CI: 0.82 - 0.94, for the interaction of distance to school and survey years 2000 and 2005 respectively).

**Figure 2:**
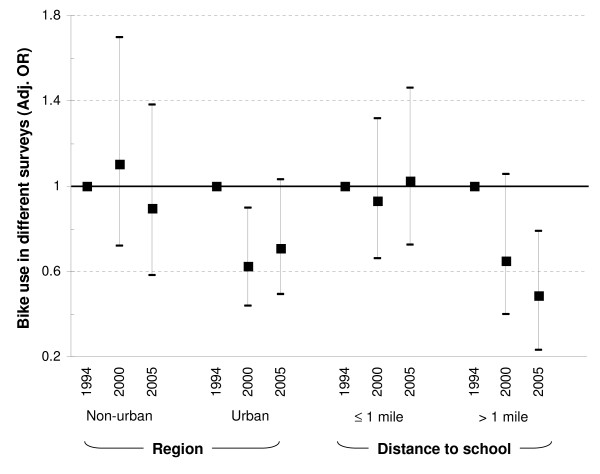
**Association between survey year and biking to school stratified by urbanisation and distance to school**. Adj. OR = odds ratios adjusted for sex, age, nationality, language, cars at home, bike availability and for distance to school or region.

Figure 2 displays the association between survey year and biking stratified by urbanisation and distance to school. In non-urban areas bike use did not significantly change over time, whereas in urban areas bike use was lower in 2000 (OR:0.63, 95%CI: 0.44 - 0.90) and 2005 (OR:0.71, 95%CI: 0.49 - 1.03) compared to 1994. Children living more than 1 mile away from school used their bikes clearly less often in 2000 (OR:0.65, 95%CI: 0.40 - 1.05) and again in 2005 (OR:0.50, 95%CI: 0.23 - 0.79) whereas this time trend was less obvious in children living closer to school.

The possibility that the high percent of children who did not have a proxy interview in the 2000 survey would influence the results was examined. There was not a statistically significant difference between children with and without a proxy interview nor an influence on the considered factors in the multiple regression models presented above.

## Discussion

This analysis of time-trends in active transportation to school (ATS) based on data of the Swiss travel survey indicate that although active commuting is still very prominent, a significant decrease in the proportion of children biking to school was noted concomitant with a significant increase in children riding a car on the way to school. Most of the changes occurred between 1994 and 2000. During the same time period significantly more cars were available per household while bike availability decreased. Distance to school did not change significantly. The decrease in bike use was more pronounced in urban areas and among children living more than 1 mile away from school and was significantly related to decreased bike availability. The increase in car use over time was not fully explained by changes associated to the number of cars per household.

The large differences in the proportion of active commuting to school between children from German-speaking as compared to French or Italian-speaking parts of the country highlights the importance of cultural factors for health relevant behaviour. The results provide a baseline for interventions of public health practitioners and urban planners.

The observed decrease in ATS in Switzerland is modest and is based on a very strict definition of active travel. The reported levels of ATS are still high when compared to those of the US [12,13], Australia [9,10], Canada [11] or the UK [14,15].

In the US, data suggest declines in ATS ranging from 48% to 13% since the late 1960 [13], in Australia from 44% to 22% since 1971 [10], in Canada from 53% to 42.5% since 1986 [11] and in the UK from 71% walking among 5-10 year olds in 1975 to 62.3% in 1989-94 [14].

Changes in distance to school have been shown to account for half of the decline in ATS in the US [13]. In Switzerland, the proportion of children living within 1 mile from school did not change significantly between 1994 and 2005 and among those who live within 1 mile from school 93.6%, 89.6%, and 90.7% actively commuted to school in 1994, 2000, and 2005, respectively. These rates are comparable to the respective proportion (86%) of US students in 1969, a number that dropped to 50% in 2001 [13]. The relatively short distances to school in Switzerland are likely to be related to the fact that more than 95% of Swiss children attend public schools and that there is no school choice. Children are usually assigned to the school located closest to their homes. In addition, factors other than distance have been shown to contribute to the choice of transport mode to school [8] including facilities to assist active travel or personal safety of the children. In Switzerland, sidewalks generally exist and 'stranger' danger is not a major concern for most of the Swiss parents [6]. Both aspects might contribute to the relatively high proportion of children actively commuting to school.

Yet, although the levels of ATS in Switzerland are less worrisome than in other countries, we note similar developments of ATS over time such as a significant decrease in biking to school and an increase in motorized transportation.

Concomitant with the decrease in bike use for travelling to school, children's easy access to bikes also decreased and when included in the analysis attenuated the time trend. Most likely, 'traffic' safety concerns of the parents which ranked high as a barrier to ATS in a previous Swiss survey [6] underlay both bike availability and bike use. The fact that the decrease in bike use occurred predominately in urban centres, a development which has also been reported from the Netherlands [19], supports this notion. From an environmental public health perspective this is unfortunate as cycling to school is a pollution-free mode of transport and provides specific health benefits as has been shown in a recent Danish study [2]. Children and adolescents who cycled to school were nearly five times more likely to be in the top quartile of cardio respiratory fitness than those who walked or travelled by motorized transport. Promoting cycling as a mode of transport to school might thus be of particular public health relevance. Yet, the observed cultural differences in bike popularity between German-speaking (more than 20% of children using bikes) and French- and Italian-speaking children (less than 10%) represents a major challenge for successful health promotion and illustrates the importance of cultural factors for the choice of transport mode.

Increasing number of cars in Swiss households contributed to the popularity of motorized transportation as mode of transport to school but did not explain the time trend completely. Choosing children's transport mode to school is an integral part of the household decision-making process [14] and includes many factors [8] such as the possibility of linking the school journey with the journey to work, convenience, parental concerns about road safety, social and cultural norms. These aspects may play an important role but were not assessed in the travel survey.

### Strength and limitations

The present analyses are based on national transport survey data which are the only available source in Switzerland which capture travel behaviour details and allow to describe the time trend in ATS in a representative Swiss sample of children. However, this routine database has obvious limitations as it provides only very limited information about factors that potentially influence the choice of children's transport mode. In addition, changes in methodology such as in the proportion of proxy interviews may introduce inaccuracies limiting interpretation of time trends. Further, it has to be noted that data on time trends were only available for the last 11 years.

## Conclusions

In conclusion policy makers and public health professionals should continue to support programs designed to encourage children's active transportation to school such as KidsWalk-to-School (Pédibus) initiatives and to increase efforts to facilitate and support the use of bikes such as safe bike-lanes. In addition, community authorities need to continue placing schools within an acceptable walking distance from the children's homes and improve road safety

## Competing interests

The authors declare that they have no competing interests.

## Authors' contributions

CB and EM contributed to the study conception and design. LG and BB obtained the data and performed the data management. LG and CB conducted statistical analyses and interpreted the data. CB and LG wrote the paper. All authors critically revised the draft versions of the manuscript, provided critical feedback and approved the final version.
